# Overcoming barriers: enhancing medical autopsy rates through effective communication and IT integration

**DOI:** 10.1007/s00428-025-04115-4

**Published:** 2025-05-10

**Authors:** Samuel Rotman, Christel Gerber, Andreas Konasch, Charline Allilaire, Pierre-Alexandre Bart

**Affiliations:** 1 Service of Clinical Pathology, Lausanne University Hospital and University of Lausanne, Lausanne, Switzerland; 2 Service of Internal Medicine, Lausanne University Hospital and University of Lausanne, Lausanne, Switzerland; 3 Direction of Information Services, Lausanne University Hospital, Lausanne, Switzerland

**Keywords:** Autopsies, Communication, Request, Digital demand, Education

## Abstract

Medical autopsy activity has been declining for several decades to the point of completely ceasing in some European countries. The reason for this decline in activity was initially attributed to advances in imaging techniques as well as the costs involved. In this study, we have attempted to demonstrate that there is a way of increasing medical autopsy activity without any additional financial input or increase in human resources. Our results showed that good communication on the usefulness of medical autopsies within the medical and paramedical population and good training on how to request a medical autopsy from the family of the deceased patient would make it possible to increase the activity of medical autopsies without additional financial input. The second part of this study attempted to demonstrate that there is potential for increasing medical autopsy activity through the development of a digital process for requesting medical autopsies, with the aim of reducing the time spent on such a request by the clinician. Taken together, our results demonstrate the importance of communication within the medical and paramedical communities and with the family of the deceased patient for the success of medical autopsies. It also highlights the facilitating effect of digitisation in the medical autopsy request process for the clinicians.

**Supplementary Information:**

The online version contains supplementary material available at 10.1007/s00428-025-04115-4.

## Introduction

In the main, two types of autopsies can be distinguished. The first type of autopsy is the medical autopsy, which is requested by clinicians after obtaining consent from the family of the deceased patient. The financial responsibility for this medical procedure is assumed by the institution, thereby relieving the family of any financial burden. The second type of autopsy is the forensic autopsy, which is conducted at the request of a judicial entity and of which the costs are borne by the losing party. In Europe, the organisation of autopsy process requests varies considerably. There are countries where no medical autopsies are conducted, and there are others where almost every death involves a forensic autopsy. Most countries that perform medical autopsies have reported a decline in their utilisation, with some countries having ceased this practice entirely. Conversely, forensic autopsies have remained a standard procedure, as required by various judicial bodies [1–3].

The principal factors responsible for this decline in activity are the high workload (time spent with the family of the deceased to convince them to accept the request for an autopsy, to summarise the medical history of a life, focus on the questions of interest to the pathologist, and to report the results to the family) and the associated costs [4, 5]. An erroneous hypothesis occasionally put forward to explain this phenomenon is that advances in imaging technologies have made medical autopsies superfluous [6]. Other erroneous assumptions, such as the suggestion that religion is a contributing factor to the reduction in medical autopsy activity, are also prevalent. However, a review of the medical literature reveals that these claims are unsubstantiated [7, 8]. One can draw parallels with the field of transplantation and organ donation. Spain, for instance, is an illustrative case study, exhibiting a strong Christian influence while simultaneously demonstrating exemplary transplantation activity at the European level. In Switzerland, the attainment of the ISFM (*Swiss Institute for Continuing Medical Education and Training*) (our national body for quality control of medical training for all doctors and certification of medical specialities) to obtain the Swiss specialist’s certificate in pathology requires the completion of 80 medical autopsies.

While there is no doubt as to the necessity of medical autopsies for the education of post-graduate medical doctors, it is important to emphasise that the activity of medical autopsy provides an excellent quality control index for clinical activity, particularly for our own institution [9]. Moreover, it is incumbent upon various public health services at the regional and national levels to regularly collate statistics on the distribution of different causes of death. In this regard, medical autopsies can be of significant utility to government statistical services, providing validated causes of death and enabling the prioritisation of certain projects or decisions. Furthermore, hospital and healthcare hygiene services can also benefit from medical autopsy activities, particularly by conducting surveys based on the results of the autopsy analyses. As the utility of medical autopsies at the hospital level is beyond question, the current trend of abandoning this activity could result in poor decision-making due to a lack of valuable indicators produced by medical autopsies.

The objective is to prevent the decline of this activity, indeed, even to stimulate its practice, by developing a policy of *promotion and communication* with all partners involved in the medical autopsy activity. An additional approach is to develop *an effective approach to communicating with families regarding medical autopsies* at the postgraduate level. The aim of this approach would be to reduce the number of refusals, thereby increasing the number of autopsies performed. In the absence of these two fundamental preliminary steps for the promotion of autopsies to our partners, it is reasonable to conclude that clinicians will be unable to make an autopsy request without a clear understanding of the rationale behind the existence of medical autopsies.

In the event of a death, it is incumbent upon clinicians to take the time to explain the *rationale* for requesting an autopsy to the family. Subsequently, a considerable amount of time must be dedicated to the completion of the autopsy request forms. It is therefore not surprising that there is a reluctance to request an autopsy, particularly during night and weekend shifts. To increase the number of medical autopsies performed, a communication project was initiated, involving all relevant stakeholders. To conduct this study at the institutional level, it was first necessary to secure the support of the Hospital General Direction. This was with a view to raising awareness among various department heads of the importance of completing a medical autopsy request for every in-hospital death. It was necessary to develop *a system of internal communication* to disseminate information to all trades related to a deceased patient, including nurses, transporters, funeral services, and clinicians. It was also necessary to implement *external communication support* for the general population to facilitate greater acceptance of medical autopsy requests by patients and their families. Conversely, the process of requesting medical autopsies is arduous, time-consuming, and undoubtedly a source of resistance, which has resulted in a decline in the number of medical autopsy requests. The project of developing a *digital medical autopsy request process* to facilitate or reduce the workload on the clinical side could therefore be a solution to increase medical autopsy activity. To achieve this objective, two prospective and successive studies were developed with the aim of increasing medical autopsy activity without increasing expenses for the hospital. The first objective is to enhance communication, and the second is to develop an electronic medical autopsy request form. The present study reports the results of an evaluation of the impact of both studies on the incidence of medical autopsies.

## Material and methods

### Human resources

The human resources dedicated to the performance of medical autopsies comprise a part-time senior pathologist (SR), a full-time junior pathologist, and two full-time autopsy technicians.

### Communication study

On the medical side, we asked all clinicians to start a mental process of reflection on the steps involved in requesting a medical autopsy for every death in our hospital. To help clinicians with that process, we identified five reasons for performing an autopsy to bring them some elements to better explain the reasons for accepting an autopsy by the family of the deceased. The communication study was conducted over a 4-year period between 2014 and 2018. The objective was to establish a set of *criteria* for communicating the process related to a medical autopsy request. Our criteria were based on the ‘S.M.A.R.T’ method: Specific, Measurable, Achievable, Relevant, and Time-bound*.*

The following five scenarios were identified as potential reasons for an autopsy:

1. Unexpected natural death
2. Death in a research protocol context
3. Evaluating the effectiveness of new treatments
4. Validation of listed clinical diagnoses
5. Family request for an autopsy

Regarding *internal communication*, a structured teaching module (3 h in duration) was devised with the objective of imparting the requisite knowledge to formulate a request for an autopsy to the deceased’s family. (additional data are given in Online Resource 1). The teaching staff for this module comprised a psychologist and a senior pathologist (SR). The teaching was proposed on a biannual basis to all clinical services. In the initial hour, the procedure for requesting an autopsy was explained in detail, followed by a discussion of the formulation of the aforementioned request. Subsequently, a role-play was conducted with the participation of actors. The participants were primarily physicians and nurses. Subsequently, the total number of teaching hours was calculated and categorised under the heading of ‘internal communication’. To reduce the taboos surrounding autopsies, an information session was held for all hospital employees every 5 to 6 weeks, with details published on the intranet website (additional data are given in Online Resource 2, 3). The dedicated time was recorded under the heading of “internal communication”.

In all services of our university hospital, an open discussion was proposed to physicians and nurses to explain the rationale behind the request for a medical autopsy and the subsequent procedure. The aforementioned information was disseminated to all services at least once every 2 years (additional data are given in Online Resource 2, 3). The dedicated time was duly recorded under the heading of “internal communication”.

In the pathology service, clinicians were afforded the opportunity to discuss the results of their analyses at their convenience. These discussions were conducted in the form of a clinicopathological correlation conference, with two to three such meetings held annually. In return, our institution provided us with the assurance that no legal measures would be taken against any employee in the event of a divergence of opinion regarding a diagnosis. The dedicated time was duly recorded under the heading of “internal communication”. To address the most frequently asked questions (FAQ) from patients or their families, the authors, with the aid of the institutional information service, created brochures and dedicated website pages (additional data are given in Online Resource 4, 5). Furthermore, the dedicated time was also recorded under the heading of “internal communication”.

Furthermore, the time spent on various presentations on this study at national and international conferences was recorded under the heading of “external communication”.

Considering funeral service providers is also a significant contributing factor to the success of our study. Indeed, during the transportation of the body, funeral service providers frequently establish close and confidential relationships with the deceased’s family. The privileged time between the funeral service providers and the deceased’s family can influence the family’s decision to perform a medical autopsy or not, based on the information they have received. Accordingly, time was allotted for communication with funeral service providers on an annual basis (additional data are given in Online Resource 6). The dedicated time was recorded under the heading of “external communication”.

To evaluate the influence of communication on the activity of medical autopsies, the number of hours recorded under the heading of “internal and external communication” was aggregated on an annual basis and compared to the autopsy rate of the service of general internal medicine (SGIM). Indeed, given that the SGIM is the largest provider of autopsy requests for the pathology service of our institution, the statistical power would thus be enhanced.

It is also noteworthy that the commitment made to various clinical services was to produce and provide autopsy reports for clinicians and the family within a timeframe of 4 to 6 weeks.

### Digital autopsy request form study

The digital autopsy request form study (DARF) was conducted over a 3-year period between 2019 and 2022. Firstly, the clinical needs were identified to ascertain the most relevant points in terms of efficiency, namely reducing resistance, reducing the development cost for IT, and ensuring ease of implementation over time. Subsequently, the requirements of the pathology service were evaluated, and a list of technical issues for IT support was compiled. The digital form was integrated into the SGIM as part of the record management system. The digital form was furnished with dynamic links (additional data are given in Online Resource 7). The transmission of requests was conducted via spreadsheet (additional data are given in Online Resource 7). The junior pathologist was required to copy and paste data into a text file to generate the final form. The data collected permitted the calculation of certain indices, namely the acceptance/refusal rate of autopsy requests and the time elapsed between the submission of the request and the autopsy.

### Statistical analysis

Statistical analysis was conducted using Stata version 18.0 (Stata corp, College Station, TX, USA). Associations were assessed using nonparametric Spearman correlation coefficients. Statistical significance was assessed for a two-sided test with *p* < 0.05.

## Results

### Communication study

The results of the communication study were evaluated according to the number of hours dedicated to this task. It can be reasonably assumed that this workload has been underestimated. Indeed, during the period under review, the senior pathologist (SR) was frequently required to respond to phone calls from numerous clinicians, take part in informal meetings, and engage in discussions with various families or physicians outside the hospital, as well as attend in-person preparations for the restitution of certain results or various court hearings as a witness. It should be noted that the evaluation of communication work does not consider its quality, since no evaluation survey was conducted after each meeting. Moreover, the impact of communication itself requires a certain time to disseminate and integrate among collaborators, not to mention the personnel turnover rate. Assuming a period of 2 to 3 years for the integration of communication, it can be observed that the rate of autopsies for the SGIM relative to the number of deaths in that service has more than doubled over the past 4 years, increasing the rate from 0.073 to 0.182 (Table 1). This is all the truer given that communication about medical autopsies was non-existent before our project.

**Table 1:** Data of communication study

Year	2012	2013	2014	2015	2016	2017	2018	2019	2020	2021	2022
Autopsies number in pathology (AP)	107	93	118	141	132	108	110	102	101	92	82
Autopsies number from internal medicine (INM)	10	5	19	6	29	31	45	41	31	24	35
Ratio INM/AP			0.16	0.04	0.22	0.29	0.41	0.4	0.31	0.26	0.43
Number of deaths in internal medicine (DIM)	278	229	260	210	247	254	246	235	331	342	332
Rate of autopsies in internal medicine (ratio INM/DIM)	0.036	0.022	0.073	0.028	0.117	0.122	0.182	0.174	0.093	0.07	0.102
Internal communication time (IHN)	0	0	81	50	63	53	63	34.5	16.5	19.5	13.8
External communication time (EHN)	0	0	11	2.5	11	11	11	21	21	21	0
Total time of communication (THN)	0	0	92	52.5	74	64	74	55.5	37.5	40.5	13.8
Nb hours of communication to gain 1 autopsy	0	0	6.5	− 4	3.2	32	5.2	− 13.8		Mean, 4.85	

We also compared the period of communication with the previous period (without communication), although the size of our cohort was too small to statistically demonstrate a difference.

**Fig. 1:**
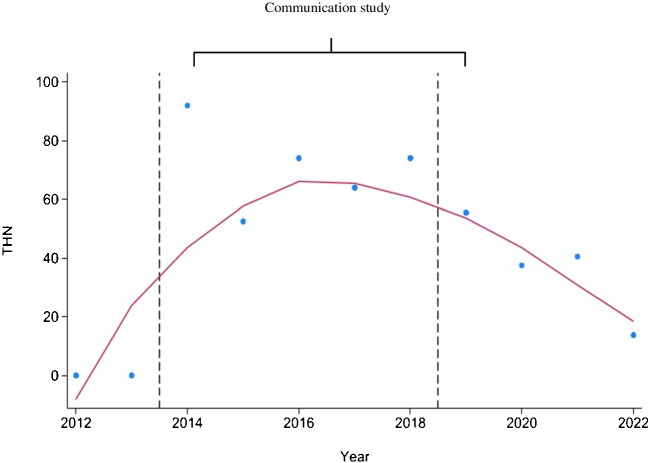
Correlation between autopsy rates and total hours of communication (THN). THN increased during the communication study, only to decrease afterwards. THN also appeared to be correlated with the rate of internal medicine autopsies: Spearman correlation coefficient 0.589, with a *p*-value at the limit of significance (*p* = 0.058)

The *impact* of communication on the autopsy rate has been evaluated by employing the autopsy rate and the number of hours dedicated to internal, external, and total communication (Table 1). The data (trendline in red line) demonstrated a progressive enhancement in communication efficacy over time, reaching its zenith at the conclusion of the study period. Total communication hours (THN) also appeared to be correlated with the rate of internal medicine autopsies (Fig. 1). Furthermore, the same analysis was conducted for the 4 subsequent years following the cessation of communication (2019–2022). The efficacy of communication on autopsy activity exhibited a decline from the second year following the cessation of the study (Fig. 1).

We then studied the effect of internal communication compared with external communication, but due to the small size of our cohort, we were not able to demonstrate any difference. It should be noted that the investment in terms of communication hours was much greater for internal communication than for external communication. Furthermore, we sought to ascertain the number of communication hours necessary to achieve one additional autopsy in comparison to the preceding year (i.e. the energy expended in communication work). The mean value of this quantity over the 4-year period is approximately 5 h of communication per additional autopsy (Table 1).

### Digital autopsy request form study

The evaluation of the effectiveness of the DARF presents a significant challenge. It is important to acknowledge that the evaluation period of this digital autopsy request study was significantly impacted by the global pandemic of Covid (Fig. 1). During Covid, hospital staff were under pressure and could no longer devote time to formulate requests to families or even to address the request to the pathology service. It was therefore not possible to statistically demonstrate the effect of the digital request on the autopsy rate. We also tested a statistical attempt to remove the Covid effect on the autopsy rate. However, the results do not allow us to draw any conclusions. Nevertheless, all the medical staff reported that they appreciated this change to this digital request, which could reduce constraints for clinicians, and had the strong impression that they would spend less time filling in the autopsy request form.

Nevertheless, the digital system enabled the observation that approximately 40% of deaths in the SGIM had not been the subject of an autopsy request, despite the requirement for such a request from the deceased’s family in accordance with the established procedure (Table 2). Moreover, the rate of acceptance of autopsy requests is relatively low, representing approximately 10% of deaths in the SGIM. One of the most probable explanations is the way an autopsy is requested from the deceased’s family (Table 2).

**Table 2:** Data of digital autopsy request form study

Year	2019	2020	2021	2022
Autopsies number in pathology (AP)	102	101	92	82
Autopsies number from internal medicine (INM)	41	31	24	35
Ratio INM/AP	0.4	0.31	0.26	0.43
Number of death in internal medicine (DIM)	235	331	342	332
Rate of autopsies in internal medicine (ratio INM/DIM)	0.17	0.09	0.07	0.10
Requested and accepted (RA)		31	24	35
Ration RA/DTH		0.15	0.08	0.12
Number of RA during day (8 h–18 h 30)		18	15	13
Day ratio of RA		0.58	0.63	0.37
Number of RA during night (18 h 31–7 h 59)		13	9	22
Night ratio of RA		0.42	0.38	0.63
Requested and refused (RR)		121	180	186
Ratio RR/DTH		0.57	0.62	0.63
Number of RR during day (8 h–18 h 30)		58	79	84
Day ratio of RR		0.48	0.44	0.45
Number of RR during night (18 h 31–7 h 59)		63	101	102
Night ratio		0.52	0.56	0.55
Number of not requested autopsies (NRA)		59	85	74
Ratio NRA/DTH		0.28	0.29	0.25

It is challenging to ascertain the acceptance rate of requests made during the day or night, given the considerable variation in rates, which range from 40 to 60% during the day (Table 2). Furthermore, the refusal rate is also challenging to evaluate, given the considerable variation between 40 and 60% between day and night (Table 2). However, these data appear to contradict the assumptions or preconceived ideas that would suggest a higher refusal rate at night than during the day.

## Discussion

Autopsy activity is declining across Europe [10]. Although initial explanations attributed the decline in autopsy activity to advances in imaging and to religious factors, contemporary research indicates that the primary causes are related to communication with the deceased’s family and the time available to make such requests to the family [11–13]. One potential solution, as proposed in the literature, is to foster postgraduate training [7–10]. Nevertheless, it is notable that few studies have sought to assess the influence of communication on autopsy activity [11].

The objective of this study was to enhance autopsy activity. To this end, two main avenues were explored: the implementation of internal and external communications and the development of a DARF.

In terms of communication, a series of information sessions were conducted for physicians, nurses, and funeral services. Written communication was disseminated to various target audiences, including collaborators, physicians, families, and patients. Additionally, a list of FAQs was made available on the institution’s website. In conclusion, a postgraduate teaching module dedicated to medical autopsy requests was implemented.

The quantification of this communication was based on counting the number of hours devoted to it, with the understanding that there is a certain degree of underestimation of the actual time spent. The quality of this communication was not evaluated using questionnaires following each information session. However, its impact on the increase in medical autopsy activity seems to play an important role, as the autopsy rate more than doubled during the period of communication. Over the last 15 years, the autopsy rate has fallen steadily. It was only when a wide-ranging and targeted communication campaign was implemented that autopsy activity was able to increase. However, the time commitment involved in conducting such communication is considerable, and it may not be feasible for a single individual to maintain this level of engagement over an extended period. Furthermore, there is a saturation effect over time, which results in a decline in the efficacy of in-person meetings at various stages and a reduction in the impact on collaborators. One potential solution would be to disseminate information through a variety of channels and formats, with the support of the human resources department and communication techniques (such as dedicated podcasts for families, collaborators, and students).

It is possible to achieve a further increase in medical autopsy activity without any problem, but this would require an increase in the number of personnel employed in the human resources department. This point has also been emphasised in European guidelines [14].

At the institutional level, this study tends to show that autopsy activity can be increased through the implementation of targeted and effective communication strategies. This underscores the importance of robust human resources and communication policies, especially to have a good quality control metric for hospital management.

Regarding the DARF study, it is challenging to draw conclusions due to the potential influence of the residual effect of our communication study on the results and the Covid period. It is noteworthy that medical activity was sustained in the absence of communication and throughout the period of the pandemic. This also serves to underscore the considerable efforts made by clinicians.

The implementation of a digital system for the request of autopsies permitted the assessment of the acceptance rate of autopsy requests conducted by clinicians. To date, no results have been produced; however, the first set of findings reveals no statistical difference in acceptance rates regardless of whether the request was made during the day or night. This highlights the significance of the contributions made by clinicians. Furthermore, the study indicates that there is a relatively high refusal rate for medical autopsies, which suggests the potential for improvement in the training of collaborators on how to request such an examination.

Overall, all clinicians expressed satisfaction with the digital request system, citing significant time savings as a key benefit.

## Conclusion

The results of this study tend to demonstrate that it is possible to increase the number of autopsies performed by improving communication and reducing the workload for clinicians. Nevertheless, it is also imperative to have hospital support policies in place to facilitate such activity.

## Supplementary Information

Below is the link to the electronic supplementary material.

ESM 1 (PDF 1.41 MB)

## Data Availability

All data generated or analysed during this study are included in this published article.
