# Supplementary material for: Overcoming barriers: enhancing medical autopsy rates through effective communication and IT integration
Source: Virchows Arch. 2025 May 10;487(4):767–73. doi: 10.1007/s00428-025-04115-4 (PMC12546307; doi:10.1007/s00428-025-04115-4)
Supplement: Supplementary file 1 — (PDF 1.41 MB) [file 428_2025_4115_MOESM1_ESM.pdf]

Overcoming Barriers: Enhancing Medical Autopsy Rates Through Effective Communication and IT Integration.

Samuel Rotman[1], Christel Gerber[2], Andreas Konasch[2], Pedro Marques-Vidal[2], Charline Allilaire[3], Pierre-Alexandre Bart[2]

1- Service of clinical pathology, Lausanne University Hospital and University of Lausanne, Lausanne, Switzerland.
2- Service of internal medicine, Lausanne University Hospital and University of Lausanne, Lausanne, Switzerland.
3- Direction of information services, Lausanne University Hospital, Lausanne, Switzerland.

Corresponding author

Samuel ROTMAN

Samuel.rotman@chuv.ch

ORCID : 0000-0002-2508-3725

Rue du Bugnon 25

1011 Lausanne

Switzerland

Online Resource 1: Requesting a medical autopsy course

Announcement of bad news: role play with actors

1. Scenario description

System(s) evaluated	Communication
Main objective of the position	*That participants practice requesting an autopsy in medical/nursing pairs, using active listening, empathy, reformulation and congruence.*
Secondary objectives of the position	Know the procedure for requesting an autopsy<br>Learning to work in an interprofessional context
Main diagnosis (if relevant)	Grade III anaplastic oligoastrocytoma in the setting of a low-grade glioma diagnosed in 2008.
comorbidities	
Context of care	Hospitalized for seizure management

3. Starting situation

Interview context :	Interview conducted in a hospital setting by a doctor and a nurse with the parents of a young patient who died.
Reasons for consultation :	Virginie's parents arrive just after their daughter's death to receive request for an autopsy.

4. Information for simulated patient recruitment

Brief summary of the case for simulated relatives

Virginie (30y) has had a brain tumor (anaplastic oligoastrocytoma grade III as part of a low-grade glioma) since the age of 25. The tumor had been contained for around 4 years by multiple operations, chemotherapy and radiotherapy, and had worsened over the last 1 year. Virginie, who had completed an apprenticeship as a laboratory technician, stayed on to live with her parents. In view of her deteriorating condition, you have been particularly attentive to her over the last few months. You have adapted the house so that Virginie can move around in a wheelchair when she is too weakened by her treatments or epileptic seizures, and for several weeks now you have been taking turns at her bedside, administering her anti-epileptic treatments. Virginie has also been suffering from severe headaches and intracranial hypertension for the past month, which are difficult to relieve with cortisone and morphine treatments.

You, the parents; Victor (self-employed accountant) and Madeleine (part-time self-employed hairdresser), are aware of the progression of the disease, but you find it hard to really contemplate your daughter's death. As for Virginie, she never talks about the end of her life. Virginie used to run track and field, then triathlon, and was a strong-willed young woman who decided not to let the disease get her down. She has many friends and keeps in touch with them through social networks. You also have a son, Jules, 2 years younger than Virgine, who lives away from home and works as a landscaper.

Virginie had to be hospitalized because you were no longer able to manage the epileptic seizures that were becoming a daily occurrence, despite her consistent anti-epileptic treatment. She had a fall during her last seizure at home, but it wasn't serious. This was very unsettling for you. You also feel that hospitalization is important to improve pain relief. You feel helpless in front of your daughter's increasing pain.

The situation in hospital is gradually deteriorating as the disease progresses. Epileptic seizures remain frequent, despite the adaptation of treatments. Virginie is becoming increasingly tired. The "do not resuscitate" decision was discussed with her and you. It was a difficult moment, as Virginie had to come to terms with her situation.

Virginie's condition has stabilized over the last 2 days, giving her hope that she'll be able to go home with you. In this context, you are going out to dinner with your son to rest together for a while. At your request, we've arranged for a volunteer to be present so that you can leave quietly, as has already been done on a few occasions.

Unfortunately, in your absence, Virginie suffers severe respiratory distress. You are informed by telephone that Virginie is having difficulty breathing and that you should return quickly to support her. By the time you arrive in the room, 15' after the phone call, Virginie has died. You are greeted by the nurse, who then leaves you alone with your daughter for a moment. On leaving the room, you wait in a small upstairs lounge for the nurse and doctor, to find out what has happened. During this interview, an autopsy will be requested. Your son hasn't arrived yet, because when he called, he had no idea that Virginie's death was imminent.

**Age around 55-60**	**Female/male**	**Build and height Indifferent**
**Clothing, make-up**	**Context (inpatient/outpatient)**<br>Inpatient	

**Emotional state of Virginie's parents**<br>You are obviously very moved by your daughter's death and feel guilty for not having been there. You don't understand what precipitated her death when she was feeling a little better, but you are realistic about the fact that your daughter was in the final stages of her disease.<br>At the interview you wanted to have with the professionals after spending some time with your deceased daughter, you don't know that a request for an autopsy is going to be made. You should have a space to talk about your emotional experience before the request is made.<br>The request comes as a surprise, because the disease is well known and your daughter has already suffered too much to suffer any more: "She's already had so many tests, so many surgeries, can't we just leave her alone? what more do you want to learn? what good will it do?"<br>It's possible that the argument of understanding what precipitated her death at a time when the situation had stabilized, can affect you, especially since you don't understand why she died of a respiratory problem. You're torn between the need to understand to relieve yourself of guilt, and the need to stop making her suffer. It's very hard for you to have to make a decision now.	
**Fears of the simulated patient**	**Expectations of the simulated patient**

OBJECTIVES FOR THE PAIR :

1. Enter into a relationship and conduct an interview to announce a diagnosis, using the acronym SPIKES[1,2].

   1- Teike Lüthi F et al. Annonce de mauvaises nouvelles : une pointe d'EPICES dans l'apprentissage. Rev Med Suisse 2011; 7 : 85-7.

   2- Baile WF et al. SPIKES – a six step protocol for delivrering bad news : Application to the patient with cancer. The Oncologist 2000;5:302-11.

2. Use the pair as a resource for caregivers and patients during the interview.

Instructions for the situation pair (a doctor and a nurse)

Virginie Glardon, born in 1985, has been hospitalized for the past two weeks in the internal medicine department for a decline in general condition and recurrent epileptic seizures due to a grade III anaplastic oligoastrocytoma within the context of a low-grade glioma diagnosed 5 years earlier. She had a minor fall during her last seizure at home, which destabilized her parents.<br>She's a sporty girl, who used to do athletics and triathlon, and is a laboratory technician. She has lots of friends and keeps in touch with them via social networks when she doesn't have too many headaches. She's full of projects. She has a brother 2 years her junior and, given her health situation, lives with her parents, who manage with their jobs (they are both self-employed) to look after their daughter.

Despite chemotherapy, radiotherapy and multiple operations, the disease is progressing and Virginie is experiencing an increase in epileptic seizures, as well as severe headaches due to intracranial hypertension. A DNR decision was discussed with her and her parents, which was not easy, as Virginie had been fighting to live for years and was not ready to contemplate the end of her life.

Treatments were adapted over the course of these 2 weeks, with little result at first: increasing asthenia, daily epileptic seizures and headaches that were difficult to relieve. Over the past 2 days, however, her condition has stabilized, she feels a little better and is now talking about organizing a return home, as she doesn't like being in hospital.

In view of this stabilization, the parents went off to eat together with their son. Not wishing to leave their daughter alone, they arranged for a volunteer to be present.

Unfortunately, during their absence, Virginie suffered severe respiratory distress. The parents were notified by telephone so that they could return promptly. By the time they arrived in the room, some 15' after the phone call, Virginie had died. They were greeted by the nurse, who left them alone with their daughter for a moment.

The nurse and doctor were present at the time of death: in the face of this terminal dyspnea, doses of Morphine and Dormicum i/v were administered in agreement with Virginie to relieve her, which was the case.

As you leave the room, the parents are waiting for you in a small upstairs lounge to take another moment with you and find out what happened. Their son hasn't arrived yet. During this new interview, you must suggest that they carry out an autopsy on Virginie. On one hand, this request is part of our desire to help them better understand what happened to Virginie and thus help them in their grieving process. On the other hand, as part of a change in institutional policy, an autopsy request must be made for every death for which the cause is unclear.

Proposed programme: 1pm-5pm

1pm: Introductions of actors and participants, expectations/intentions.

Setting the framework: objectives, rules (confidentiality, respect, etc.), pairing, programme

1h30 pm: Theory reminders

- Stress
- Conduct of interviews
- SPIKES
- Autopsy (definition, legal, pathological, etc.)

2h-2h15 pm: First scenario

- Presentation of the actors
- Reading of the vignette
- Choice of pair
- Preparation of everyone (actors and pairs on their own and participants in pairs, SPIKES)
- Instructions to participants (who they observe - actors/carers, what they note as good and a suggestion for constructive improvement for a particular aspect)
- Role playing: 10 minutes
- Freeze frame: how do they feel?
- Feedback from carers - then from actors - then from participants
- Debriefing, discussion, questions-theoretical contributions, etc.

3h15 pm: Break

3h15-3h40 pm: Second scenario

- - Same procedure as first scenario

4h45 pm: Evaluation – close.

Online Resource 2: slide set

New autopsy request procedures

Dr S. Rotman, IPA
Project manager

Context

Current indications for requesting a clinical autopsy

Unexpected death

Death in the context of a research protocol

Assessing response to treatment

At the family's request

Necessity of autopsy activity

For services

Establishing cause of death
Clinical correlation (treatment efficacy, imaging, etc.)

For quality control

Diagnostic errors despite imaging =>
The uselessness of autopsies is a false argument

For social and preventive medicine

Monitoring the quality of death certificate results => statistics

For Hospital Hygiene

Identification of undetected infections at the time of patient handling =>.
Influence on other patients, caregivers, family and friends, infection detection and quality of care

Success factors

For pathologists

Provide an information procedure to respond to the request (reasonable and defined deadlines)
Set up a codified autopsy register to provide indicators of activity (number of requests, rate per death, discordance, etc.)
Adapting autopsy examination methods and technological developments
Organize regular colloquia with the main requesting departments

For CHUV

Clearly communicate policy internally and externally
Guarantee employees of the institution's support in the event of consequences linked the data of the deceased patient's relatives (diagnostic error)

In practical terms

In the event of death at the CHUV

Determine the cause of death according to procedure:
Action to be taken in the event of patient death and autopsies

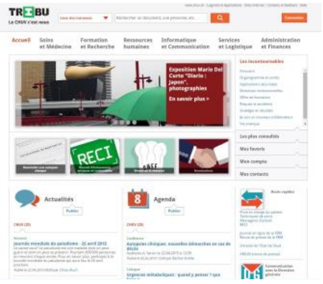

In practical terms

In the event of death at the CHUV

Even if the autopsy is forensic, a request for a clinical autopsy must be made to the next of kin!

In practical terms

Requesting an autopsy for all deaths at the CHUV

Family agreement

The request must be made with conviction by an MC and/or CDC
- Explain the reasons for and usefulness of this last gesture
- Always accept a request from the family.

MAs can attend, provided they have taken the "Bad news module" course.

If the family ***agrees***

- Attach signed agreement to patient file

If the family ***refuses***

- Note name of requesting physician, date/time and reason for refusal

In practical terms

Autopsy request for all deaths at the CHUV

Announcement in Pathology

Medical

Application form (after consulting Directive CHUV sur Décès)
- Internet: autopsy and chuv
- download...
- Send completed form by e-mail
- Notify the pathologist

At the nursing level

- Patient ID sheet
- Care techniques
- Organize patient transport to the IPA chapel
- Call the chapel manager

In practical terms

Autopsy request for all deaths at the CHUV

Deadlines for results

The next day
- by telephone: macroscopic appearance

A 10j
- 1er report: macroscopy

At 1 month
- Complementary/final report (if no CNS): microscopy and clinical correlation

SNC
- Final report: CNS, microscopy and clinical correlation

In practical terms

Restart of:

- Systematic discussions with departments, taking their needs into account

Conclusions

Autopsy is essential for training in pathology.

Autopsy is vital for clinician training and research.

The inclusion of systematic medical autopsy in the CHUV's quality policy is supported by management and financed by the IPA's current resources.

It provides a final check and closes the quality loop.

It is useful in view of diagnostic discrepancies.

It is an important issue for all partners in the healthcare system, and particularly in a training institution.

Online Resource 3: Frequently asked questions (FAQ) for the clinicians (translated from French).

1. What autopsies are carried out over an extended weekend?

For **adult autopsies**, the autopsy request and the body must be received by the Service of Clinical Pathology before 9am on Saturday morning. Any autopsy request received after this time will be proceed on the next on-call day in the event of a long weekend. Please note that autopsies suspected of containing prions (Creutzfeld-Jacobs disease) are carried out by the neuropathologist on the next working day. **Paediatric autopsies**, where the cause of death is likely to be a metabolic or infectious disease, are considered as urgent. We will then carry out an immediate autopsy. Please notify the on-call pathologist, who will then contact the paediatric pathologist. For other causes of death, paediatric autopsies will be carried out on the next working day.

2. What happens with bodies whose autopsy is carried out on the next working day?

If the death occurs at University Hospital of Lausanne, Switzerland, the body remains in the Hospital's cold room during opening hours. If the death occurs in an external hospital, the body is placed in the hospital's cold room or transferred to pathology, depending on the opening hours of the Hospital’s chapel. If death occurs at home, transfer the body to the Hospital chapel.

3. Who will pay for the autopsy?

Autopsy is a free service, except for foetuses under 22 weeks, which will be charged on the same basis as a surgical specimen.

4. When is the chapel open?

Monday to Saturday : 8.15am-11am and 12.30pm-5pm

Sundays and public holidays:8.15am-12pm and 1pm-3pm

The personal of the chapel will answer to your questions.

5. What do I do if I have any doubts about whether an autopsy should be carried out by forensic medicine?

If the death has occurred at the CHUV. The Institutional procedures should be applied.

If the death occurred outside the CHUV:

Notify the prosecutor or call the forensic pathologist on duty:

6. What are the different options for taking care of the body after a paediatric autopsy?

If the family takes the body back for burial, it will be made available in the Hospital’s chapel after the autopsy. In this case, the parents must contact the funeral directors of their choice directly, who will take charge of the body.

If the family chooses to leave the body at our Service of Clinical Pathology, it will be incinerated at the funeral centre at the expense of the City of Lausanne and the ashes will be placed in the cemetery's garden of remembrance (families must be informed that in this case there will be no name or recognition number).

If the family wishes to have the ashes returned, this is only possible for residents of Lausanne or if the birth took place at the University . For other districts, the choice is to take back the body and have it incinerated by a funeral company at the expense of the family.

7. How are samples taken for cytogenetic and bacteriological placental tests?

For cytogenetic tests, a sample is taken from the skin of the heel (0.5 cm2) and placed in a culture liquid or 0.9% NaCl. The whole sample is placed in the fridge. For cytogenetic tests, parental authorisation is required.

For bacteriological examinations of the placenta, the foetal surface around the umbilical cord must be sterilised by cauterisation (burning with a scalpel), then the amniotic surface must be incised and a swab passed in a circular movement through the subchorionic fibrin under the membranes. Place the smear in the fridge.

8. How can I send a sample to the Service of Clinical Pathology outside opening hours and during weekends?

For urgent analysis, use the pneumatic line from the operating room. If equipment needs to be dropped off at the Service of Clinical Pathology, call the Security team who will open the doors to drop off the fixed sample.

9. Is there an on-call service for pleural fluids, ascites, BAL and CSF during the weekend?

A cytotechnician is always present on Saturday mornings until midday, except for CSFs. From 5pm to 8am and during weekends, an on-call service is provided by the central haematology laboratory**.** This may be requested for CSF, which must be analysed as quickly as possible.

Online Resource 4: Frequently asked questions (FAQ) for the general population (translated from French).

1. The medical autopsy

Patients come to hospital hoping to be cured or at least treated. Although this hope is more and more frequently realised these days, some patients do succumb to their diseases. While their loved ones are in mourning, they may have many questions about the cause of death, or whether the events occurred as their doctors explained to them. An autopsy can help relatives to understand what happened and to ease their grief. It can also uncover or clarify the diagnosis of genetic diseases that may have consequences for the deceased's descendants. But the autopsy is also the only way of monitoring the quality of care and treatment provided by the clinic and is an important support for teaching and research. An autopsy is therefore crucial not only for the clinic, teaching and research, but also for the family.

2. What is a medical autopsy?

A medical autopsy is not a forensic autopsy. It only concerns deaths resulting from natural causes. A medical autopsy can only be carried out with the agreement of the family. Its purpose is to clarify all clinical diagnoses, to assess the response to treatment and to search for the cause of natural death. They also enable radiological images (radiology, prenatal ultrasound, etc.) to be correlated and compared with autopsy findings. They are also useful for correlating with surgical or medical interventions (status of anastomoses, assessment of the function of certain organs, etc…). This quality control is vital for improving our clinical facilities, but also for dealing with innovative procedures (transplants, minimally invasive surgery, interventional radiology, etc.), resistant infections or new pathologies (SARS, etc.). To improve communication with the family when an autopsy is requested, a brochure entitled 'What is a medical autopsy?' can be printed from our website.

In some respects, an autopsy can be compared to a surgical operation. It involves a thorough external and internal examination. The autopsy is performed by a certified specialist in pathology. This means that the doctor is specially trained for this type of examination. The autopsy normally lasts a few hours. It is conducted with the greatest respect and dignity towards the deceased. The incisions are made discreetly and are well hidden by clothing. The organs are removed from the body and inspected with the naked eye. During the discretion by the pathologist, small fragments of tissue are removed for later examination under a microscope. An attempt is then made to answer the questions raised by the family and the clinical doctors. For example, is there a metabolic disease, an infection, or even a malignant tumour? Is there a neurological disease, such as Alzheimer's? What was the cause of death? Is there a hereditary disease? A genetic examination is also possible, such as a search for pollutants or occupational diseases.

3. Which organs are removed?

An autopsy is an analysis of all the body's organs, including the brain. A fragment of each organ is removed. The heart is removed for in-depth, systematic analysis. Brain removal may be refused if the family refuses or for aesthetic reasons.

4. What are the consequences of an autopsy?

After the autopsy, everything will be done to leave as few traces as possible, so that the body can be presented to the family in the best possible conditions. In reality, there will be an abdominal and thoracic scar, which will be perfectly sutured by our trained staff. The chapel staff will complete the aesthetic aspect for presentation to the family.

In the case of analysis of the central nervous system, every aesthetic means will be used to hide the scar and allow the body to be presented to the family. The results are excellent.

5. What are the reasons for an autopsy?

There may be several different reasons, but in general, it is requested by the doctors treating the patient to monitor the quality of their care, to check the response to treatment or to clarify a diagnosis or the cause of death. There may also be a request from the family, when they want answers to their questions. This kind of

motivation greatly helps families to get over the bereavement phase and helps to resolve certain ambiguities with the treating doctors, particularly when the contract of trust has been broken. The pathologist-physicians will then do their utmost to provide as many answers as possible to all these questions.

It is not uncommon that an autopsy makes fortuitous discoveries which, depending on the diagnosis, could have medical consequences for the family of the deceased.

6. Who is informed of the results of the medical autopsy?

The results of examinations following a medical autopsy are distributed to the requesting doctor, with a copy to his or her department head. A copy of these results is also sent to all treating doctors during the patient's lifetime. In the event of a breach of the contract of trust with the attending doctor, or simply in accordance with the family's wishes, a copy will be sent to the doctor of the family's choice.

The pathologist will not discuss the results with the family. Nor will the results be sent directly to the family. However, all the results can be discussed with the family at the doctor of their choice.

7. How long will the results take?

An initial result based solely on macroscopic observations is communicated orally to the requesting doctor within 48 hours after the autopsy. Within 10 days, a written report of this macroscopy is sent to all the doctors.

If the autopsy does not include an analysis of the central nervous system (CNS), a final report is sent within one month. If the autopsy includes an analysis of the CNS, a supplementary report will be sent which will include the conclusions of the microscopic analysis. Approximately 3-4 months after the autopsy, the final report with the conclusions of the CNS analysis will be sent to all the doctors.

8. What advantages does the family gain from a medical autopsy?

The autopsy provides answers to the family's remaining questions. This process is an important step in the grieving process. The pathologists will do everything in their power to answer to them, and in particular to provide explanations or determine the cause of death. In the event of a breach of trust, requesting an autopsy helps to re-establish a climate of trust with the attending physician or hospital. It can also be used to investigate certain genetic diseases that may have consequences for the deceased's family.

9. How do you request an autopsy?

The requesting doctor goes to the website of the Service of clinical pathology from our University Hospital of Lausanne, Switzerland.

Select the ‘autopsy request’ tab and fill in the form in digital ‘word’ format. Once he has completed the form, he sends it to us by e-mail. The request does not need to be signed by hand. The identity of the requesting doctor on the e-mail and on the request is sufficient. He or she then notifies the pathologist by telephone.

Transport of the body is then organised with the staff of the Pathology Chapel.

Online Resource 5: Leaflet for the general population (translated from French).

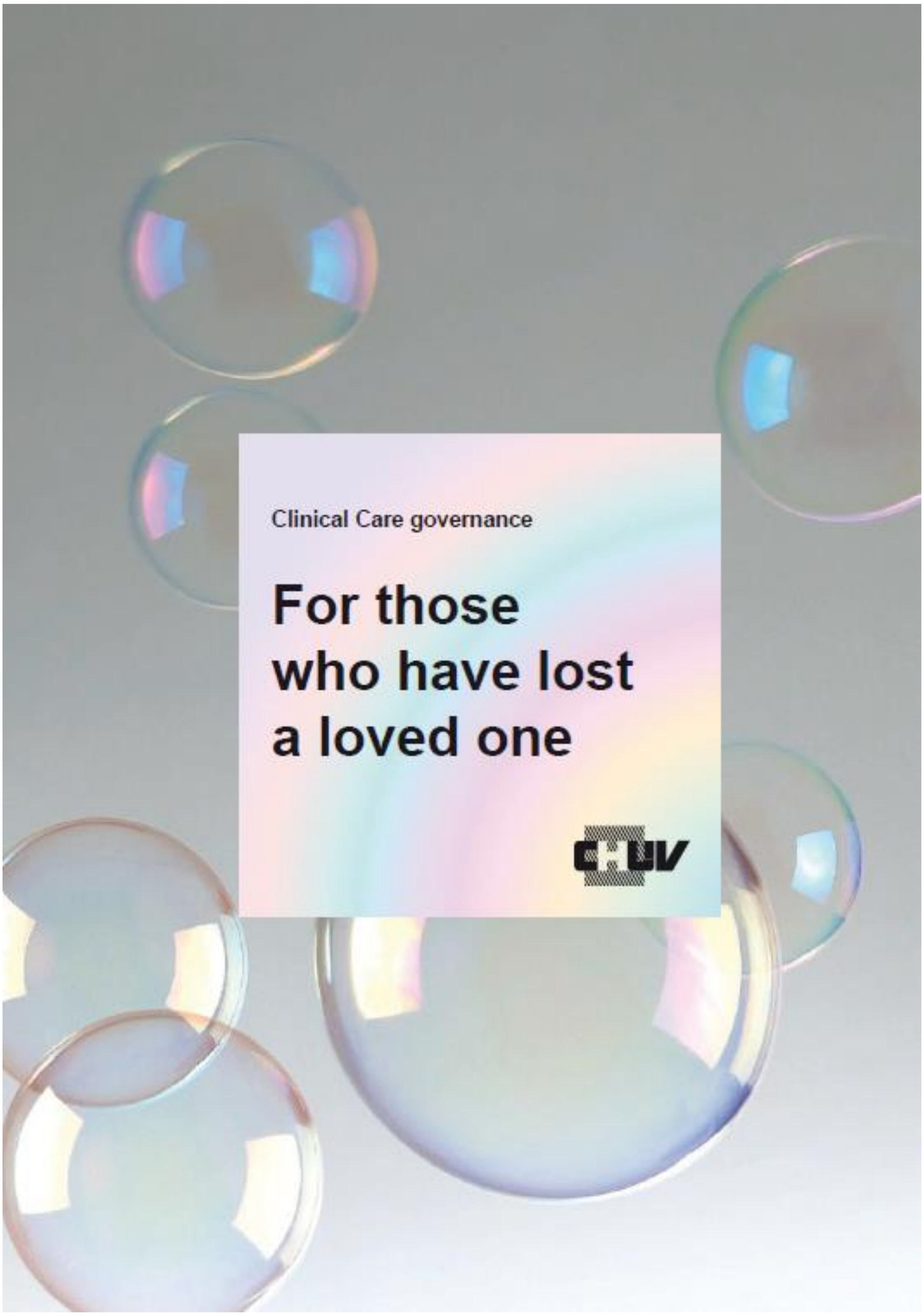

Dear Madam, Dear Sir,

You've just lost someone close

and we would like to offer you our sincere condolences.

This brochure is intended

to make administrative procedures easier in the event of death.

IN SUMMARY

In the care unit

Where and when to visit a deceased loved one? What about a request for an autopsy?
What about corneal donation?
What can the Hospital's ecumenical chaplaincy offer you?

The effects of the deceased

Where can I bring clothes for the deceased?
Where can I find the deceased's personal effects? Where to collect valuables?

Administrative procedures

How do I prepare for the funeral? What other steps should I take? How can I get help?

After hospital

How to organize a religious ceremony? How do I arrange for the deceased to be transported abroad?
Where can flowers be delivered?
Where can I find support?

IN THE CARE UNIT

Where and when to visit a deceased loved one?

In general, in the hours following death, you can visit the deceased in the care unit where he or she spent his or her last moments.
The care teams there provide you with the support and information you need. Before being taken care by the funeral director of your choice, the deceased is transferred to the mortuary chapel, where visits are also possible. Parking is available on site, and we recommend that you call ahead.

Thereafter, the deceased will be transferred to the location of your choice by the company.

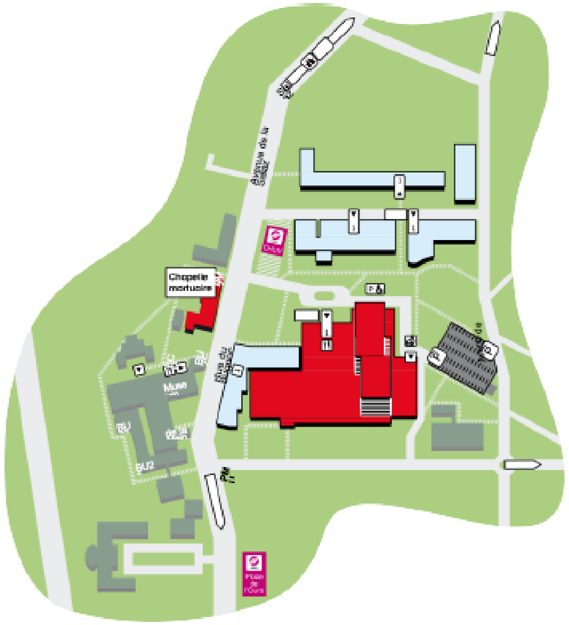

HOSPITAL COMPLEX

What about a request for an autopsy?

You may be asked to perform a medical autopsy.

It's a difficult request to make at such a painful time. But it's too important to pass over in silence, and you should know that the family has the right to object to this medical autopsy.

Don't hesitate to ask questions before you make your decision.

An autopsy is an important medical procedure, as it helps us to understand the illness from which the deceased was suffering, as well as the cause of death. Sometimes, it also helps to advance medical knowledge and treatments

It removes any doubt and can be a source of comfort for you.

The autopsy results are forwarded to the doctor who requested it (generally the doctor who attended your loved one) who in turn will send them to the attending physician, from whom you can obtain the information you require. There is no charge for an autopsy, and it generally does not interfere with funeral arrangements.

In exceptional cases (e.g. following an accident), the judicial authorities may request a forensic autopsy; you cannot oppose to this request.
The body is then transferred to the forensic center of Lausanne and you must inform the funeral director so that they can contact this center.

In this case, and if you wish, you can ask the forensic center of Lausanne to arrange for an appointment.

The forensic doctor will explain the results of the autopsy to you, with the agreement of the authority that requested the forensic examination.

What about corneal donation?

Corneal donation can be carried out after death, and health professionals may ask for your consent.

The cornea a transparent window in front of the color of the eye. Opacification leads to permanent loss of vision or blindness if both eyes are affected.
In these cases, the only possible treatment is to replace the cornea with a transplant.

Although not widely publicized, corneal donation meets a real demand, and some 400 corneal transplants are carried out every year in Switzerland. Despite this, there is a shortage of donors. In French-speaking area of Switzerland, 140 patients are constantly waiting for a transplant, sometimes for 2 to 3 years. During this time, their quality of life is diminished, and it is sometimes impossible for them to work.

The removal of corneas requires the agreement of the donor or his or her next of kin. The wishes of the deceased take precedence over those of the next of kin. If the deceased's wishes are not known, the next of kin must make the decision on his behalf, while respecting his presumed wishes.

The sample is taken with all due respect, and no marks are visible on the deceased's face. It must be carried out as soon as possible after the death and not interfere with the funeral rites.

Donation is anonymous, free of charge and does not entail any additional costs for relatives. For further information, please contact the Eye Bank at the Jules-Gonin Eye Hospital in Lausanne.

What can the Hospital's ecumenical chaplaincy offer you?

A chaplain is available at all times to accompany hospitalized patients and their families on their spiritual and/or religious journey.
Don't hesitate to call on him during the time of separation and farewell that you were experiencing at the time of death. At your request, the nurse on duty can contact the chaplain on duty.

THE DECEASED'S PERSONAL EFFECTS

Where can I bring clothes for the deceased?

At the Hospital mortuary chapel. You can also deliver them directly to the funeral office of your choice.

Where can I find the deceased's personal effects?

Clothes, small sums of money and objects of lesser value are kept in the care unit for around four days until they can be handed over to the family.
You can then pick them up at the reception desk at the Hospital's main entrance.

Where can I pick up my valuables?

The Hospital will send valuable items (money deposited by the patient on arrival at the Hospital's main cashier or kept in the care unit, valuable jewelry, etc) directly to the judicial unit.

For further information, please contact Secretariat.

How to prepare for a funeral

The Hospital will hand over the death certificate to the funeral director you have appointed, who will take charge of the body and gather the necessary documents.

If you wish, the mortician can also organize the funeral: transportation of the deceased, announcements, religious services, etc. You can also take care of these formalities yourself. In this case, we invite you to take the following steps:

1 / At the Hospital

At the mortuary chapel, we will deliver to you a necessary document for further steps called *death certificate* (several copies).

2 / Lausanne Civil Registry Office

You will need to obtain a ***death certificate.*** The procedure is the same, whatever your district of residence. This step is essential, and you must provide the following documents so that the death can be registered and the appropriate action taken:

- ❑ death certificate
- ❑ family record book or marriage certificate
- ❑ proof of address
- ❑ for single people: individual civil status certificate
- ❑ for foreign nationals: a copy of an identity card, residence permit, birth certificate if divorced or single
- ❑ for widower' death: certificate of spouse

3 / Burial and cremation office

The office will issue a ***burial or cremation*** for you.
to the commune of the deceased's destination. Here, too, the procedure is the same, whatever your commune of residence. On site, you'll need to present :

- ❑ death certificate
- ❑ the death certificate issued to you by the Registrar's Office

For further information, visit the City of Lausanne website or contact the Burial and Cremation Office directly.

What other steps should I take?

Once the death has been registered, the Civil Registry will notify the following official bodies:

- The AVS fund (via AVS head office in Geneva)
- The judicial office of the district in which the deceased resides
- The district income tax office of the district of the deceased
- The district origin of the deceased
- The relevant Consulate when necessary
- The guardianship authority if the deceased had minor children

However, it's up to you to notify your employer, bank, insurance company, etc., of your decision. You will then need to provide a *death certificate* several times. This can be obtained from your chosen funeral home or ordered directly from the Lausanne Civil Registry. Always keep the original, which will be sent to you 7 to 10 days after your order.

Please note that some insurance documents (e.g. life insurance, leasing) may need to be completed by the doctor who attended your deceased loved one at the Hospital. You can send your request and the documents by registered mail to the Hospital.

If you wish, your insurer or bank can provide you with a practical guide to matrimonial law, inheritance law and the beneficiary clause. If the deceased had drawn up a will, this document must be submitted to the Judicial instance.

How can I get help?

If you are experiencing financial difficulties, don't hesitate to contact us to inform funeral company of your choice.

For advice or problems encountered at the time of death, you can contact the Hospital social service or the social worker you met during your loved one's stay. The social service of the Ligue vaudoise contre le cancer can also help you if you have had prior contact with them.

AFTER HOSPITAL

How do you organize a religious ceremony?

You can organize the ceremony within 48 to 120 hours. This deadline can be modified by obtaining a declaration of delay or advancement. For further information, please the funeral director of your choice.

Contact your parish or religious community to organize the ceremony. This can also be done by the funeral office, if you wish. If you do not belong to a religious community, it is also possible to carry out a secular mourning ritual adapted to your wishes, in chapels of reverence or in a funeral office.
If you are planning a wake at home, you will need to make arrangements with the funeral.

Where can I find support?

Experiencing bereavement is one of life's most painful events. It's not unusual to feel deeply affected or to go through an intense procession of emotions following the loss of a loved one.
Getting back into balance can take time, and it's sometimes a good idea to get some help to get there.

You may, for example, benefit from support in bereavement groups organized by palliative care units, associations such as the Ligue vaudoise contre le cancer, or medical-social centers. If you feel you can't cope, or have symptoms that worry you, speak to your doctor.

How do I arrange for the deceased to be transported abroad?

The funeral director in Switzerland will be able to advise you and arrange transport. Alternatively, you contact an undertaker of your choice in the country of destination.

Where can I have flowers delivered?

The place where the flowers are to be delivered must be agreed with the funeral director.

PERSONAL NOTES :

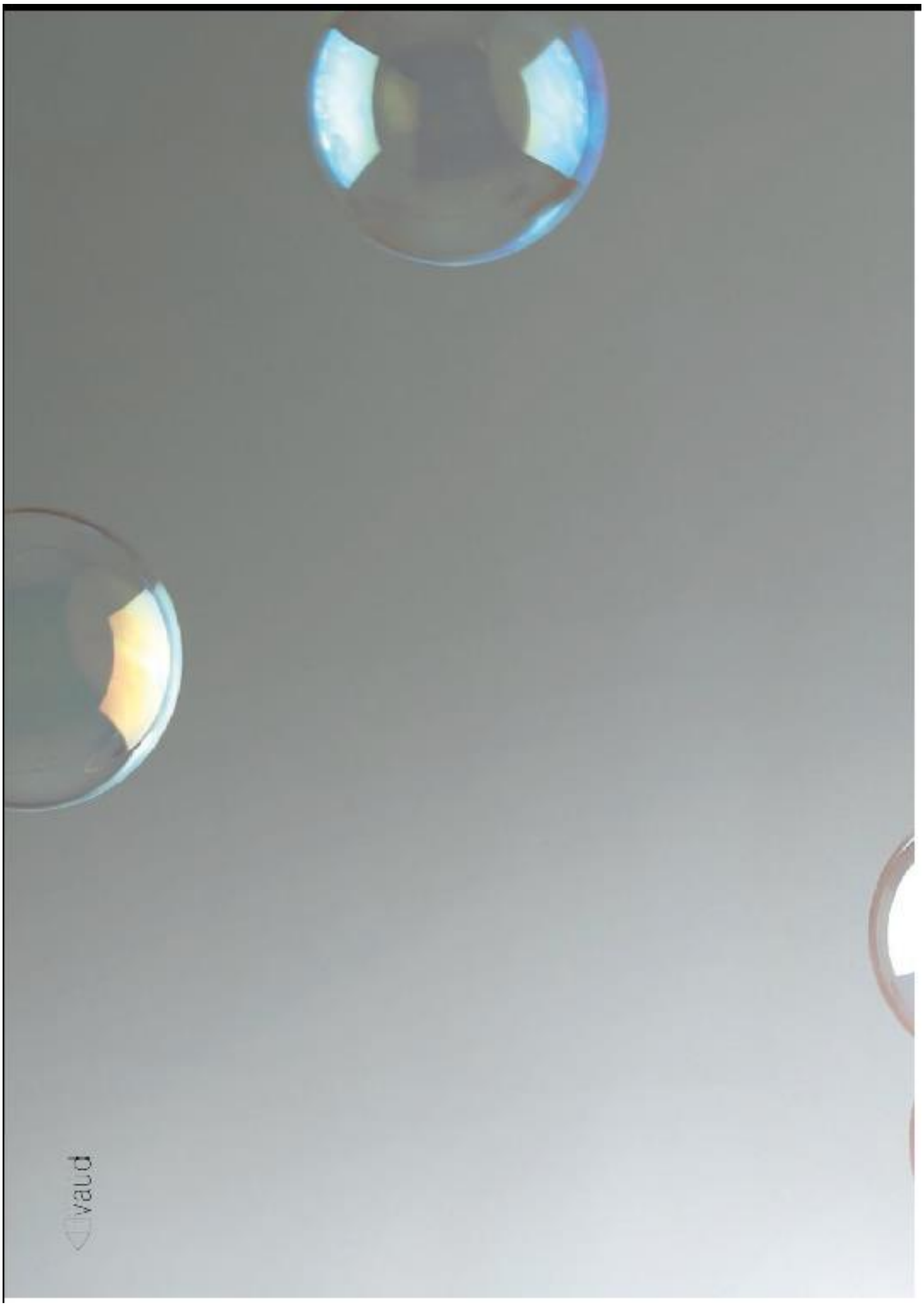
vaud

Online Resource 6: information for funeral services

In our practice?

Families feel they must pay for this ultimate gesture

Families believe they will not be able to see the body after the autopsy

The body is not always brought to the right place

Administrative delays

Difficulty reaching the right person

Confusion with forensic medicine

Confusion with body donation

Unawareness of response times and process

CHUV Institut universitaire de pathologie

Know what you're talking about!

Autopsy
Forensic autopsy
Medical autopsy
Donating your body to science
Autopsy fees
Transport costs
Death certificate
Attorney
Medical examiner
Pathologist
Forensic medicine
Pathology
Chapel
Availability of the body to the family
Examination response times

CHUV Institut universitaire de pathologie

Once a year, a meeting with all funeral services of our region has been organized. The purpose was to limit as much as possible wrong or false information delivered to the family of the deceased.

Online Resource 7: Digital form to request a medical autopsy

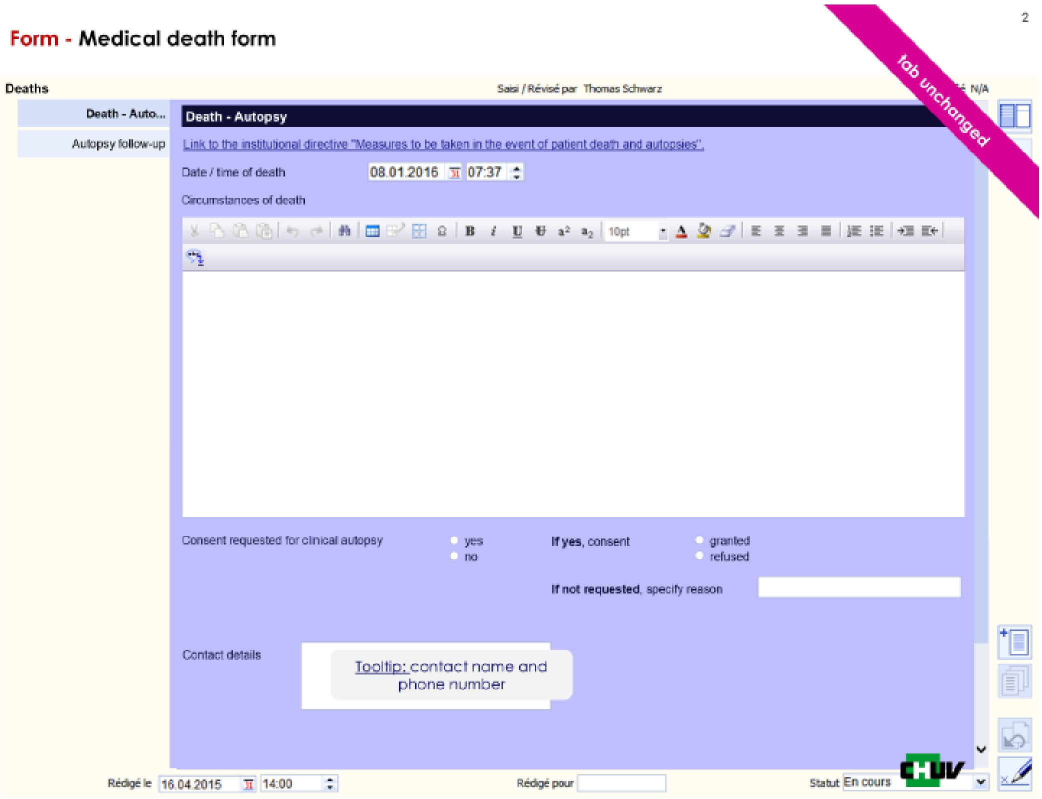

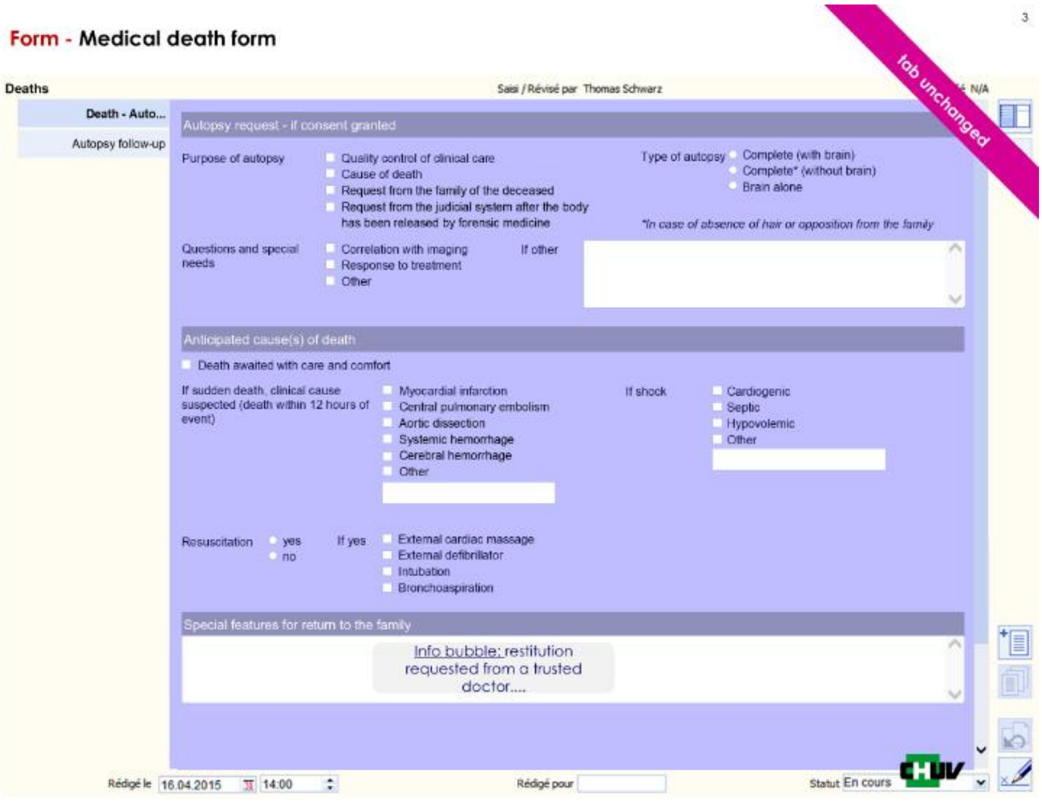

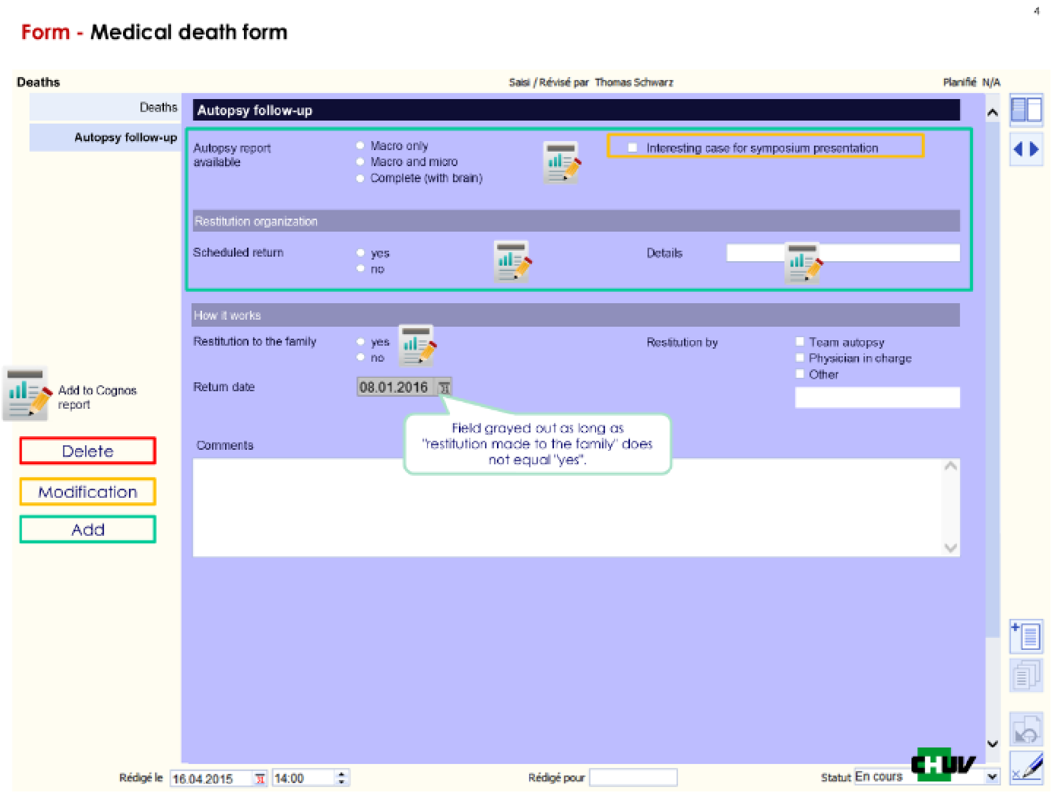

The clinician fills in the digital form. The date and time of the request will make it possible to assess the possible impact on the rate of autopsy requests. The management of the restitution of results to the family is improved by automatically scheduling an appointment according to the date of distribution of the autopsy reports.

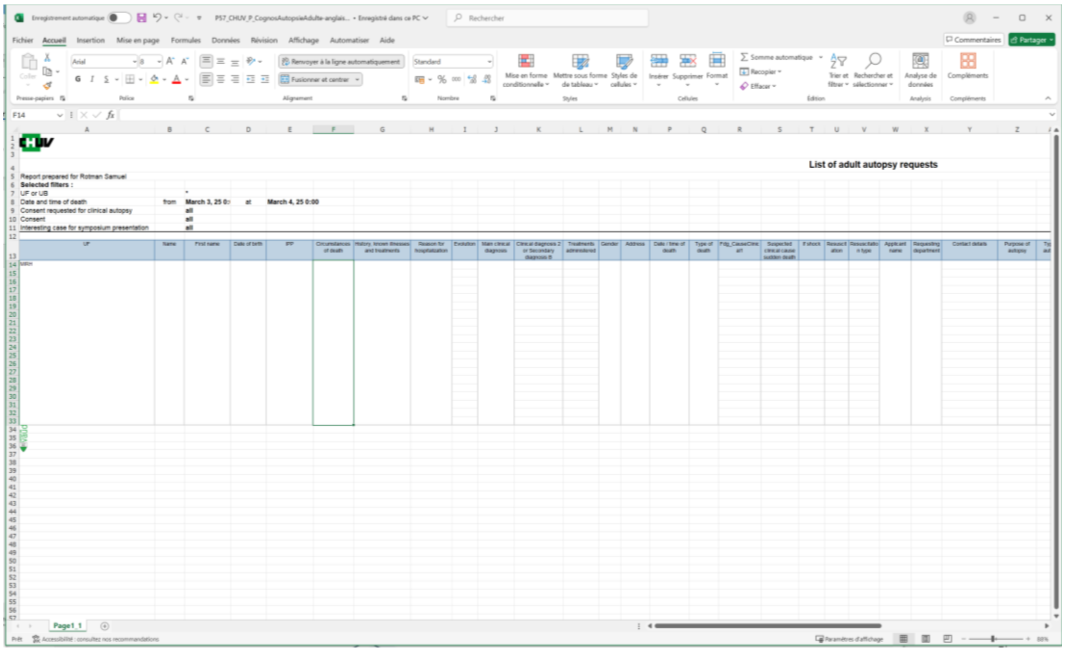

The request for a medical autopsy is automatically and digitally forwarded to the pathologist in the form of an Excel spreadsheet stored on a secure server. The pathologist can then import these data into their report.
